# phyddle: software for phylogenetic model exploration with deep learning

**DOI:** 10.1101/2024.08.06.606717

**Published:** 2024-08-08

**Authors:** Michael J. Landis, Ammon Thompson

**Affiliations:** 1 Department of Biology, Washington University, St. Louis, MO, 63110, USA; 2 Participant in an education program sponsored by U.S. Department of Defense (DOD)

**Keywords:** phylogenetic models, estimation, deep learning, neural network, supervised learning

## Abstract

Many realistic phylogenetic models lack tractable likelihood functions, prohibiting their use with standard inference methods. We present phyddle, a pipeline-based toolkit for performing phylogenetic modeling tasks using likelihood-free deep learning approaches. phyddle coordinates modeling tasks through five analysis steps (*Simulate*, *Format*, *Train*, *Estimate*, and *Plot*) that transform raw phylogenetic datasets as input into numerical and visualized model-based output. Benchmarks show that phyddle accurately performs a range of inference tasks, such as estimating macroevolutionary parameters, selecting among continuous trait evolution models, and passing coverage tests for epidemiological models, even for models that lack tractable likelihoods. phyddle has a flexible command-line interface, making it easy to integrate deep learning approaches for phylogenetics into research workflows. Learn more about phyddle at https://phyddle.org.

## Introduction

Good phylogenetic model design balances biological, statistical, and computational considerations (Felsenstein 1985b; Hansen and Martins 1996; Kelchner and Thomas 2007; Rodrigue and Philippe 2010; Servedio et al. 2014; Rolland et al. 2023). Not only should a phylogenetic model realistically describe how evolutionary lineages change across generations, it should allow accurate estimates to be obtained in a short amount of time.

Unfortunately, in practice, models with desirable biological or statistical properties often yield intractable likelihood functions. Researchers are forced to compromise: either use tractable but suboptimal phylogenetic models, or invest substantial resources and effort to design and validate boutique inference methods for more-realistic models. Most would rather not compromise at all, but must, in part due to our field’s reliance on likelihood-based inference for model-fitting. Likelihood-free approaches might be useful for model exploration, where deep learning approaches have demonstrated the ability to solve a variety of difficult, foundational problems in phylogenetics (Suvorov et al. 2020; Borowiec et al. 2022; Voznica et al. 2022; Smith and Hahn 2023; Lambert et al. 2023; Kong et al. 2023; Thompson et al. 2024; Nesterenko et al. 2024; Silvestro et al. 2024; Mo et al. 2024).

This application note introduces phyddle, a phylogenetic modeling framework for training neural networks with simulated data through supervised learning (LeCun et al. 2015; Goodfellow et al. 2016; Paszke et al. 2019). A primary goal of phyddle is to equip biologists with a deep learning pipeline workflow so they may explore and apply realistic, but otherwise intractable, phylogenetic models in the study empirical of problems. phyddle generalizes existing deep learning techniques for modeling lineage diversification and trait evolution to a broader class of user-defined phylogenetic models and scenarios (Bokma 2006; Voznica et al. 2022; Lambert et al. 2023; Thompson et al. 2024).

## Overview

**Figure 1:**
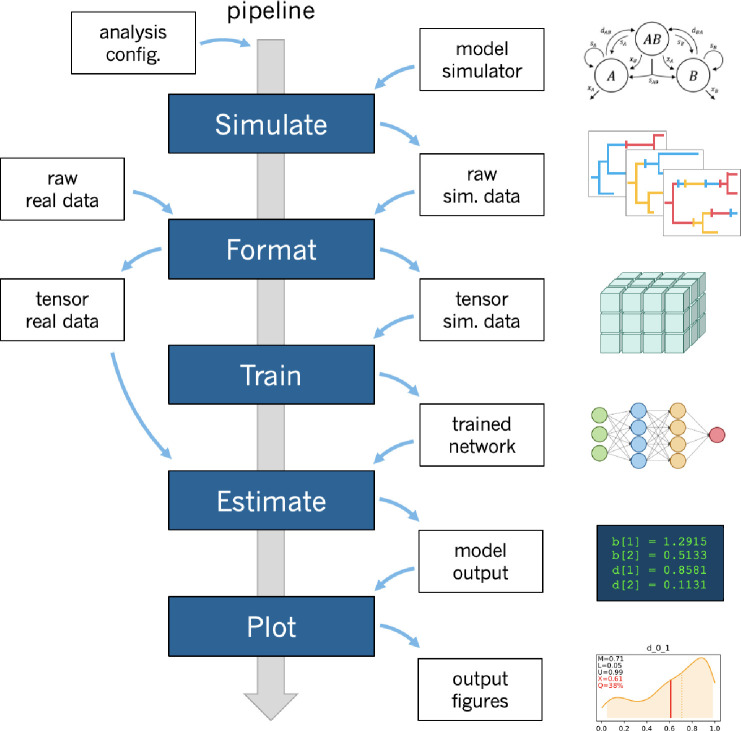
Overview of phyddle workflow. Researchers provide the software with a configuration file and a simulation script that determine the analysis settings. Upon running phyddle, the *Simulate* step produces a raw (unformatted) pool of training datasets that represent data-generating parameters (labels) and realizations of the data-generating model (examples). Next, the *Format* step restructures the raw training data into tensors, while also reclassifying simulating conditions (e.g. parameters) as either data (treated as observed) or labels (to be predicted). The *Train* step loads the tensor data, constructs and fits neural network architecture, saves a copy of the trained network, and reports training results. The *Estimate* step generates model predictions for test and empirical datasets that were not used during training. Lastly, the *Plot* step produces figures and reports that summarize the pipeline results.

phyddle uses deep learning and simulation-trained neural networks to perform phylogenetic modeling tasks. This overview assumes some basic familiarity with deep learning concepts (see Borowiec et al. 2022 or Goodfellow et al. 2016 for background reading). phyddle pipelines are composed of five modular steps (Figure 1): *Simulate*, *Format*, *Train*, *Estimate*, and *Plot*. Settings for each step are controlled through command-line options and a customizable configuration file. Being a pipeline, phyddle output is stored in a predictable manner, allowing the output from one step to become the input for downstream steps. phyddle functions as both an interactive command-line tool and as a scriptable interface, allowing researchers to design and debug workflows line-by-line before writing scripts to automate repetitive and large-scale jobs.

The *Simulate* step generates large numbers of example datasets under a model with a user-specified simulation script. We have attempted to make the *Simulate* as flexible but simple as possible so researchers may focus on model design. Users provide phyddle with model simulators written in whatever programming language suits their needs. Users are encouraged to either modify the simulator scripts bundled with phyddle, written using R (R Core Team 2013), Python (Van Rossum and Drake 2009), RevBayes (Höhna et al. 2016), MASTER (Vaughan and Drummond 2013), and PhyloJunction (Mendes and Landis 2023), or to write their own script from scratch using a language of their choice.

Each simulation script itself defines the phylogenetic model, simulates data under that model, and saves that data using standard file formats (e.g. Newick, Nexus, and/or data tables) required by phyddle. When run, a simulation generates both a training example (e.g. a phylogenetic tree and a character matrix) and the training labels (e.g. the data-generating parameters). All gathered simulations comprise the raw and unformatted training dataset. Simulation tasks may be run in batches and across processors, in parallel.

Next, the *Format* step encodes raw datasets into a set of tensors, analogous to *N*-dimensional arrays of variables, which are later processed by the *Train* step. *Format* processes all valid examples from *Simulate*, plus any empirical datasets flagged for analysis by phyddle. *Format* converts each raw dataset into three types of tensors: a phylogenetic data tensor, an auxiliary data tensor, and a label tensor. Tensors can be saved either in human-readable .csv format or in a compact .hdf5 format that is often 20x smaller than its .csv counterpart.

Phylogenetic data tensors encode phylogenetic and tip-state data into 2D tensor structure suitable for supervised learning with convolutional neural network (CNN) layers. phyddle supports the compact bijective ladderized vector (CBLV; Voznica et al. 2022) for serially sampled trees and the compact diversity vector (CDV; Lambert et al. 2023) for extant-only trees, with the option to include additional rows of phylogenetic branch length information. phyddle also allows users to associate multiple categorical or numerical traits with each taxon (+S; Thompson et al. 2024). True to the name, these encodings have a small memory/storage footprint that is linearly proportional to taxon count (compact) and uniquely identify the encoded tree (bijective) (Voznica et al. 2022).

Auxiliary data tensors and label tensors are simpler, and used as input for dense feed-forward neural network (FFNN) layers. Each row in the auxiliary data tensor represents a single, raw dataset, with each column corresponding to a different summary statistic (e.g. tree height, tree balance, etc.) or “known” model parameter (e.g. population size, sampling effort) for the data-generating process. Each row in the label tensor also references a single, raw dataset with columns corresponding to different model parameters. Labels can have numerical (rates, ancestral trait) or categorical values (model-type, ancestral state).

The *Train* step constructs, trains, calibrates, and saves a neural network and its training history. Neural networks are built and trained using PyTorch (Paszke et al. 2019) with the option to use CPU and Nvidia GPU (CUDA) parallelization. phyddle combines CNN and FFNN layers for modeling tasks (see Goodfellow et al. 2016 for review). Phylogenetic data tensors are processed as input using a set of convolutional and pooling (CNN) layers, whereas the auxiliary data tensors are processed with fully-connected, dense (FFNN) layers (Voznica et al. 2022; Lambert et al. 2023; Thompson et al. 2024). The output of these CNN and FFNN layers are then concatenated and passed into a final series of dense FFNN layers for each training target that terminate with an output layer for prediction. phyddle allows users to adjust the depths and widths of layers, convolutional settings (stride, dilation, etc.), activation functions, loss functions, and optimizer through the configuration file. *Train* uses the held-out calibration data to generate conformalized prediction intervals (CPIs) for the trained network (Romano et al. 2019) whereby e.g. an 80% CPI is the interval that has a 80% chance of containing the true parameter value, based on the known accuracy of training dataset predictions.

Supervised learning with training examples from *Simulate* is used to train, validate, and calibrate prediction intervals from the neural network. When complete, the trained network is saved to file in .hdf5 so it may be used for future estimation tasks. The training history of network loss, accuracy, etc. for both the training and validation datasets is recorded in a .csv file. Tables of predicted versus true values for the training and test datasets are also saved in .csv files. Lastly, phyddle stores the means and standard deviations needed to normalize new biological datasets for use with the trained network for predictions.

The *Estimate* step uses the trained network to make predictions from new datasets. When called, *Estimate* runs against a subset of the simulated examples that were not used during training (the test dataset) to assess performance of the trained network. *Estimate* also runs against any empirical datasets identified by the analysis. It must be stressed that *Estimate* is the simplest and fastest step, generally producing new estimates from the trained network within milliseconds. After the initial cost of generating a training dataset and training the network, new estimates from a trained network are effectively free.

Lastly, the *Plot* step visualizes all output from a phyddle analysis, beginning with empirical results and ending with network performance and architecture. Empirical estimates for numerical and categorical variables are first shown independently, and also plotted in reference against all the marginal densities and dimension-reduced joint densities of the simulated auxiliary data and labels. Network accuracy for training and test datasets are then displayed as scatter plots for numerical labels and confusion matrices for categorical labels. To help assess poor network performance, loss scores across training epochs for training and validation datasets and network architecture are shown last. In addition, *Plot* records data about a phyddle run into an easily parsed .csv file.

The modular design of the phyddle pipeline makes it relatively easy to design, train, save, and share new models with other biologists. Only a configuration file and the contents of the *Train* directory are needed for other researchers to perform *Estimate* tasks with the trained network on their empirical datasets. Entire project workspaces can be archived to ensure research is reproducible.

To showcase the flexibility and jump-start the adoption of phyddle, it is bundled with example configuration files, simulation scripts, and pipeline results for a variety of phylogenetic models (https://github.com/mlandis/phyddle/workspace). Current examples include state-dependent speciation-extinction models (SSE), susceptible-infectious-recovered models (SIR), and trait evolution models that variously use R, Python, RevBayes (Höhna et al. 2016), PhyloJunction (Mendes and Landis 2023), and MASTER (Vaughan and Drummond 2013) to simulate training datasets. These generic examples are meant to be adapted by biologists developing customized phyddle pipelines to model their systems.

## Example analyses

We applied phyddle to three phylogenetic modeling scenarios to illustrate how the deep learning software behaves relative to existing likelihood-based methods. Unless otherwise stated, phyddle analyses used default settings for the network configuration and training. The full simulated dataset was split into training (70%), validation (5%), test (5%) and calibration (20%) data subsets. Networks were trained for up to 200 epochs, but stopped early if validation loss scores worsened across three consecutive epochs. Results from the *Plot* step were reviewed to confirm each network was properly trained. Method comparisons used examples from the test data subset.

### Macroevolution under BiSSE

We simulated phylogenetic datasets under a simple binary state-dependent speciation-extinction (BiSSE; Maddison et al. 2007). The BiSSE model is a tree-generating process that assumes that speciation rates and extinction rates depend on the state of an evolving binary character. Our simulation used the following settings:

**Table:** 

$\lambda_{1},\lambda_{2}∼Unif(0,\;1)$	(birth rates)
$\mu ∼\mathrm{Unif}\left(0,\;1\right)$	(death rates)
$q∼\mathrm{Unif}\left(0,\;1\right)$	(state transition rates)
$T_{max}∼\mathrm{Unif}\left(1,\;100\right)$	(max. time)
$M_{max}∼Unif(10,\;5000)$	(max. num. taxa)

where $\lambda_{1}$ and $\lambda_{2}$ are state-dependent birth rates for lineages in states 1 and 2 respectively, μ is the state-independent death rate, and q is the symmetric transition rate between states 1 and 2. Each simulation was terminated after $T_{max}$ units of evolutionary time or when the phylogeny contained $M_{max}$ extant taxa, whichever condition arose first. Only extant taxa are retained.

We used the R packages ape (Paradis and Schliep 2019) and castor (Louca and Doebeli 2018) to simulate and fit phylogenies and character matrices with a BiSSE model. We simulated datasets with the castor::simulate_dsse() function. Datasets with 500 or fewer taxa included all taxa and had sampling fractions of 1, whereas larger trees were downsampled to 500 taxa and recorded as having sampling fractions of less than 1. All data-generating parameters and the sampling fraction were log-transformed before being saved to file for the example dataset. Evolutionary rates were treated as estimation targets, while the sampling fraction was treated as a known parameter. Simulated trees were saved in Newick format, and data matrices were stored as comma-separated value tables. This simulation procedure is stored in the file sim_bisse.R, as referenced in the phyddle configuration file below.

Maximum likelihood estimates (MLEs) for each dataset were obtained using the castor::fit_musse() function under the nlminb optimization method. To improve optimization success, each MLE represents 10 trials with 30 initial guesses (“scouts”) per trial and one “noisy” guess that is centered on the true data-generating parameters. Optimization was further restricted to search for parameter values within the simulated bounds (minimum 0, maximum 1), as it improved MLE parameter accuracy. We also expect MLE might mildly underperform in this analysis, because the inference model conditions on survival of the tree, rather than the $T_{max}$ and $M_{max}$ stopping criteria used for the simulating model.

Neural network estimates were generated with phyddle, using the config file shown as Listing 1. As the comments explain, this config expects extant-only trees with one character and two states per taxon. Four numerical parameters (log-transformed $\lambda_{1},\;\lambda_{2},\;\mu$, and q) are estimated, while one numerical parameter (log-transformed sampling proportion, ρ) is treated as known, as stated earlier. Default phyddle settings are used for any unspecified settings in the config file (see documentation).

**Listing 1:**
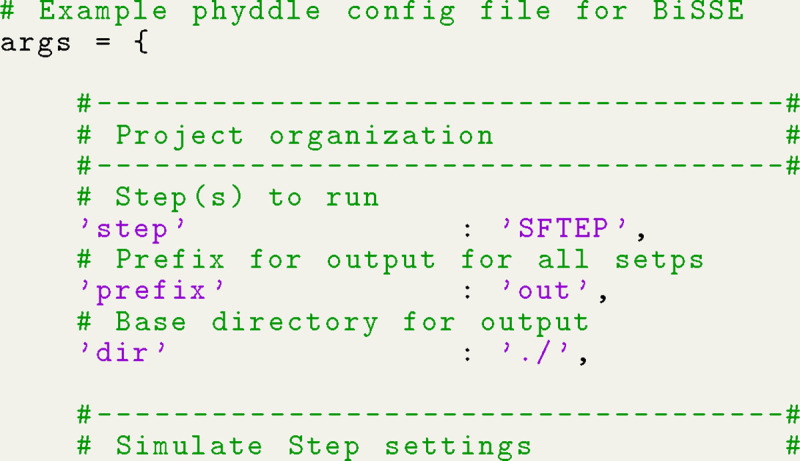

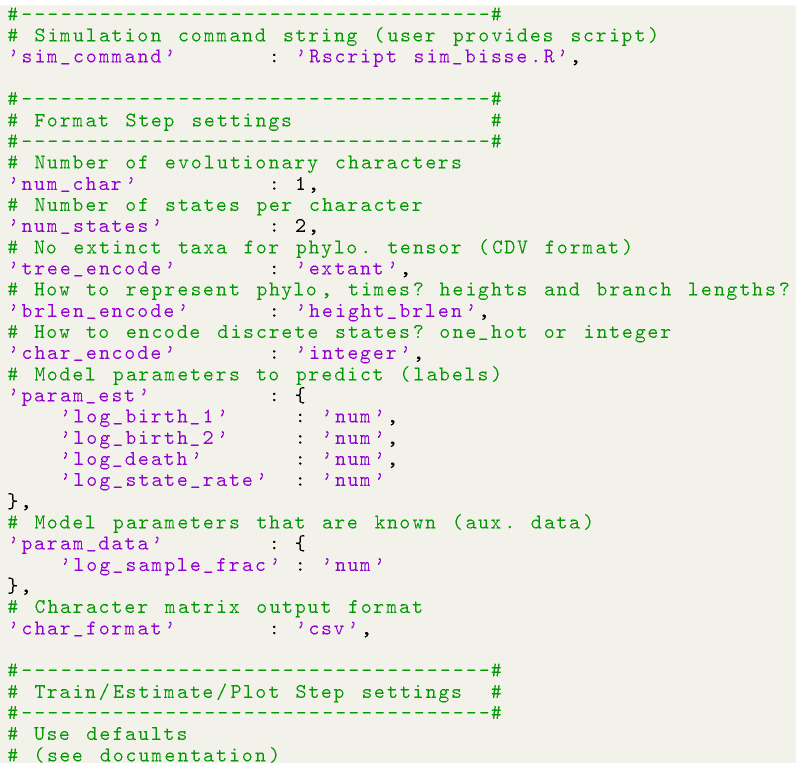
Minimal phyddle config file for BiSSE analysis. We specify only a few key settings and let phyddle apply default values to the remaining settings (see documentation).

We simulated 50,000 example datasets for the phyddle analysis. The configuration file, simulation script, and pipeline results are stored in the project archive, bisse_project.tar.gz. Pipeline results can be reproduced from scratch with the commands in Listing 2:

**Listing 2:**
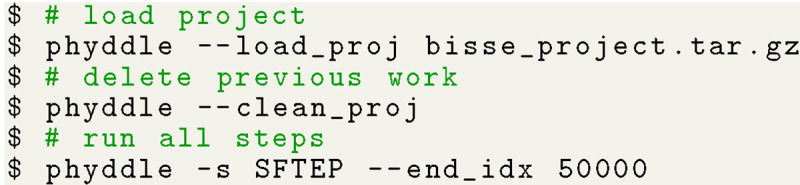
Shell commands for BiSSE analysis using phyddle.

**Figure 2:**
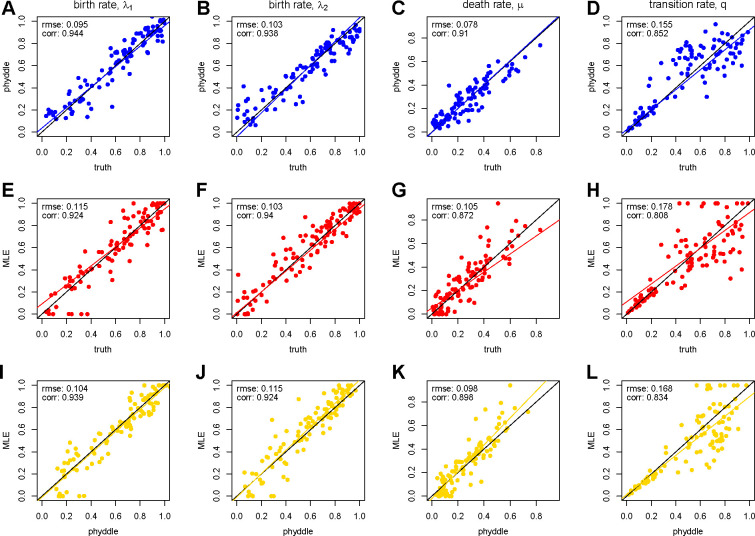
Comparison of maximum likelihood and phyddle parameter estimates. Each column corresponds to a BiSSE model rate that was estimate: $\lambda_{1},\;\lambda_{2},\;\mu$, and q. The first row compares phyddle estimates to true parameter values (blue, A-D), the second row compares MLEs to true values (red, E-H), and the third row compares phyddle and MLE estimates to each other (gold, I-L). The black line is set to intercept-0 and slope-1, representing perfectly matching values. The colored lines are standard linear regressions, and align with the black line when estimates agree with the truth (blue and red) or with each other (gold).

Parameter estimates for phyddle (Fig. 2A–D) and maximum likelihood (Fig. 2E–H) are each highly correlated with the true data-generating parameters. In addition, phyddle and maximum likelihood methods tend to agree with each other as much as the truth (Fig. 2I–L), implying both methods extract similar information from the same data pattern.

### Model selection of evolutionary mode

We simulated datasets under four models that represent alternate modes of continuous trait evolution: Brownian motion for incremental change (BM; Felsenstein 1985a), Ornstein-Uhlenbeck for stationarity (OU; Hansen and Martins 1996), Early Burst for explosive change (EB; Harmon et al. 2010), and a Normal Inverse Gaussian Lévy process for pulsed change (LP; Landis and Schraiber 2017). Trees were simulated with a standard constant-rate birth-death model, under the following conditions

**Table:** 

λ∼Unif(1,5)	(birth rate)
μ∼Unif(0,λ)	(death rate)
$T_{max}∼Unif(1,\;5)$	(max. sim. time)
$M_{min}=100$	(min. num. sampled taxa)
$M_{max}∼Unif(100,\;300)$	(max. num. taxa)

Next, for each tree, we first sampled a model type and its corresponding model parameters and then simulating the dataset:

**Table:** 

C∼{BM,OU,EB,LP}	(model type)
$X_{0}∼Norm(0,\;2)$	(starting trait value)
$\sigma^{2}∼Gamma(1,\;2)$	(process variance)
s∼Gamma(5,100)	(tip noise)
$t_{1/2}∼Gamma(4,\;2)$	(half-life; OU only)
β∼Gamma(2,2)	(decay; EB only)
κ∼Gamma(3,2)	(process kurtosis; LP only)

where the mean of Gamma(α,β) equals α/β. Process variance and kurtosis are the expected moments for trait change after one unit of time. For the OU process, process variance refers to the Brownian variance (i.e., not the stationary variance) and the optimal value is assumed to equal the ancestral value, $X_{0}$. Phylogenetic half-life relates to the OU mean-reversion strength as $\alpha =ln(2)/t_{1/2}$ (Hansen 1997). Process variance and kurtosis are reparameterized into standard model parameters for LP (Landis and Schraiber 2017). Note, these ranges of parameters were chosen so that datasets generated by more-complex models (OU, EB, LP) might be misinterpreted as originating from the simpler model (BM), thereby complicating model selection.

Trait datasets were simulated and fitted using the R package pulsR. Maximum likelihood estimates were obtained after 5 independent Nelder-Mead optimization attempts. We then computed Akiake Information Criteria (AIC) scores for each model as $AIC(c)=-2\left(L_{c}-K_{c}\right)$, where $L_{c}$ is the maximum log-likelihood and $K_{c}$ is the number of free parameters for model c. We treated the model with the lowest AIC(c) value as the model selected by maximum likelihood (Akaike 1973).

To train phyddle, we simulated 200,000 datasets with a maximum tree width of 300. Training targets included $\sigma^{2}$, κ, and C. Other data-generating parameters (e.g. $\lambda ,\;\mu ,\;X_{0}$, etc.) were treated as nuisance parameters and not estimated. We used CDV+S encoding with one row of numerical values for each species trait. We used a soft-max layer with a cross-entropy loss function to transform network output that model type C=c with probability $w_{c}$. Interpreting $w_{c}$ as proportional to the maximum (unlogged-) likelihood score for model c, we computed a penalized model selection score $SEL(c)=-2\left(ln\left(w_{c}\right)-K_{c}\right)$. We then treated the model with the smallest value of SEL(c) as the model selected by phyddle.

**Figure 3:**
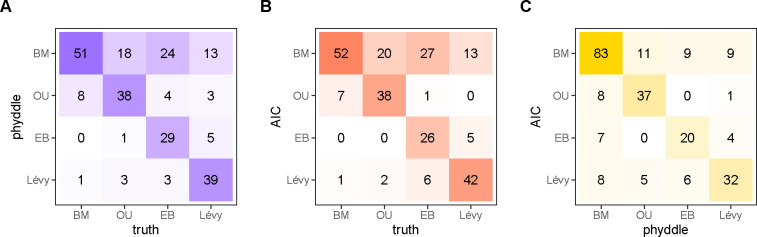
Comparison of model selection for AIC and phyddle estimates. Possible models are Brownian motion (BM), Ornstein-Uhlenbeck (OU), Early Burst (EB), and a Lévy process (LP; normal inverse-Gaussian). phyddle (A, blue) and AIC (B, red) reliably identify to true data-generating model, or the two methods agree (C, gold), when the frequencies along the diagonal are high. Off-diagonal entries represent model misidentification (A,B) or classification disagreement (C) for that column.

In general, we find both phyddle and AIC with pulsR are comparable at model selection (Fig. 3). Both methods consistently select either the true model (diagonal) or the simpler nested model (BM, top row) in the majority of cases (Fig. 3A–B). Regardless of whether they selected the true model, both methods tended to select the same model (Fig. 3C, diagonal). We note that pulsR requires the numerical integration of a recursively constructed characteristic function for model fitting, which grows prohibitively expensive for larger trees (see Landis and Schraiber 2017). The results for the full phyddle pipeline took approximately 15 minutes, whereas the pulsR pipeline took 5 hours.

### Phylodynamics under SIR with Migration

We compared phyddle to the performance of likelihood-based inference methods under two Susceptible-Infected-Recovered (SIR) model scenarios. First, we simulated data for a phylogeographic model of pathogen infection that we refer to as a SIR + Migration or SIRM model (Riley 2007). We consider the case from (Thompson et al. 2024) where all pathogen sampling occurs during the exponential growth phase early in an outbreak, accomplished by rejecting simulations where more than 5% of susceptible individuals were infected. Only during the exponential growth phase, our SIRM model behaves as a serially sampled multitype birth-death model (Stadler and Bonhoeffer 2013; Kühnert et al. 2016), and is analyzed with a known and tractable likelihood function (Maddison et al. 2007; May and Meyer 2024). We used the test data and Bayesian estimates from (Thompson et al. 2024) to compare against our phyddle estimates.

We simulated outbreaks among five locations under the following conditions

**Table:** 

$N=10^{6}$	(num. of hosts in each location)
$R_{0}∼Unif(2,\;8)$	(basic reproduction number)
γ∼Unif(0.01,0.05)	(recovery rate)
δ∼Unif((0.0001,0.005)	(sampling rate)
m∼Unif(0.0001,0.005)	(migration rate)
T=100	(sim. time)
$M_{max}∼Unif(20,\;499)$	(max. num. of sampled taxa)
ρ=0.01	(probability of extant sampled)

The per-host infection rate during the exponential-growth phase of an outbreak is $\beta =R_{0}(\gamma +\delta )$. All simulations ran for T units of time, with each contagious individual at the end of the simulation (extant) having ρ probability of being sampled. The trees were downsampled to a maximum of $M_{max}$ taxa.

In the second scenario, pathogens are sampled at a random time during the outbreak, both during and after the exponential growth phase; for this scenario we lack a simple likelihood-based inference strategy. We simulated training and test data under the same settings as above except

$$N=10^{4}$$

T=50

The smaller population size ensures many of the simulations extend beyond the exponential phase.

Note, the modeled scenarios are analogous, except that the data sampling procedure is expanded under the second scenario. We separately simulate data and train neural networks with phyddle for each scenario. We are not aware of an exact likelihood-based solution for the general SIRM model, i.e. where individuals can be sampled at any time during the outbreak and exact numbers of susceptible and infected individuals in location i at time t inform the local, instantaneous infection rate (but see Kühnert et al. 2016 and Müller et al. 2019). We emphasize that our comparison seeks to measure the accuracy of phyddle for a model with no simple likelihood-based counterpart. So, even if the unrestricted SIRM model had easily computable likelihoods, it would be trivial to engineer a more-realistic but less-tractable model (e.g. Moshiri et al. 2019; Shchur et al. 2022; Gavotte and Frutos 2022; Xu et al. 2024). As such, both our Bayesian analyses use the simpler, tractable, and time-constant SIRM model that assumes we only sample during the exponential growth phase.

We used RevBayes (Höhna et al. 2016) with the TensorPhylo plugin (May and Meyer 2024) to estimate the joint posterior density of SIRM model parameters. We assumed the population size (N), recovery rate (γ) and extant sampling probability (ρ) were known empirically, while the reproduction number ($R_{0}$), sampling rate (δ), and migration rate (m) were estimated. Prior distributions match the simulating distributions for all parameters. Markov chain Monte Carlo was run for 7,500 generations. A burnin period of 10% of generations was run before MCMC sampling and all model parameters had effective sample sizes of > 100.

We used phyddle to simulate 53,416 valid training examples for the exponential phase dataset and 96,555 for the all-phases dataset, as described above. Calibration datasets for performing conformal prediction by adjusting inner quantile estimates consisted of 6,677 for the exponential phase dataset and 37,136 for the all-phases dataset. Parameters N and γ were treated as known, whereas $R_{0},\;\delta$, and m were inferred as free parameters. We used a CBLV+S tensor with one-hot encoding to represent phylogenetic and tip states across 5 locations.

**Figure 4:**
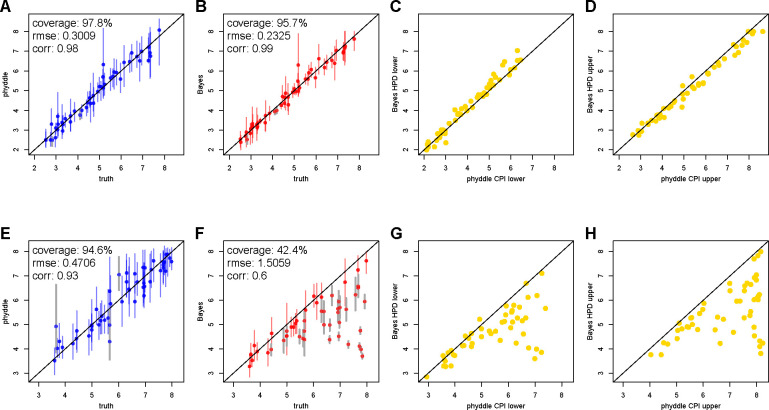
Comparison of Bayesian and phyddle estimates for the basic reproduction number, *R*_0_, when all pathogens are sampled during the exponential growth phase of an outbreak (A-D) or sampled at any time during an outbreak (E - H). True parameter values are plotted against phyddle (blue; A and E) and Bayesian (red; B and F) point estimates. Estimated support interval bounds (gold; C, D, G, and H) for phyddle and Bayesian methods are also plotted against each other. Any point that falls on a slope-1 intercept-0 line has perfectly matching *x* and *y* values. Data displayed is a random subsample of 50 values (roughly 50%). Intervals shown are 95% CPI (conformalized prediction interval) or HPD (highest posterior density). Bayesian estimates and test data for comparison of exponential phase data (A-D) are from (Thompson et al. 2024).

Under the first scenario without model misspecification for the Bayesian approach, we see that both Bayesian and phyddle point estimates for the basic reproduction number are highly correlated with the true data-generating parameter values (Fig. 4A–B). In addition, lower and upper bounds for 95% highest posterior density credible intervals and conformalized prediction intervals are tightly correlated (Fig. 4C–D).

Under the full outbreak scenario, phyddle accurately estimates the true data-generating parameters (Fig. 4E), but the Bayesian posterior point estimates and support interval bounds no longer agree with phyddle (Fig. 4F–H). That is, as the outbreak progresses, the number of new hosts that are still susceptible to infection decreases so the infection rate slows, hence the consistent underestimation for the reproductive number when the model assumes unbounded, exponential growth in the number of infections.

Similar results for the sampling rate, δ, and migration rate, m, are provided in the Supplement. This behavior is expected because, although the neural networks trained by phyddle conform to all model assumptions encoded into the simulator, the simulator violates the simplifying model assumptions required to obtain a tractable likelihood for our Bayesian analysis.

Although this experiment only demonstrates how inference methods behave when models are correctly versus incorrectly specified for two epidemiological scenarios, the broader need to mitigate model misspecification and potential solution offered by likelihood-free methods, such as phyddle, are more general.

## Conclusion

We expect that phyddle has many possible uses for researchers, both green and seasoned, applied and theoretical. We name a few ideal use cases. First, phyddle allows biologists to design and fit phylogenetic models with no known, tractable inference methods, which increases community-level access to newer models worthy of attention. Second, phyddle enables users to rapidly test whether a dataset contains any signal under standard models that have likelihood-based inference methods. A one-day analysis could inform a biologist whether they should spend a year collecting data and/or developing new methods. Third, phyddle scales well for repetitive tasks, making it ideal for analyses involving many clades, many genes, and/or assessing model sensitivity to phylogenetic uncertainty. Fourth, phyddle can be used to provide baseline estimates when developing new likelihood-based approach with no existing points of comparison to benchmark performance. Lastly, because deep learning in phylogenetics is currently underexplored, phyddle allows methods developers to systematically document how various modeling, network architecture, and training conditions influence the effectiveness of deep learning approaches. We believe these attributes will make phyddle a useful addition to the nascent field of phylogenetic deep learning.

## Software

phyddle is an open source project that is written in Python. It depends on a large number of scientific computing libraries, including: pytorch (Paszke et al. 2019), dendropy (Sukumaran and Holder 2010), numpy (Harris et al. 2020), scipy (Virtanen et al. 2020), scikit-learn (Pedregosa et al. 2011), pandas (pandas development team 2020), h5py (Collette 2013), and matplotlib (Hunter 2007). Code for phyddle is hosted at https://github.com/mlandis/phyddle. Documentation and tutorials for phyddle are hosted at https://phyddle.org. Saved workspace projects for the three examples in this manuscript are hosted at https://github.com/mlandis/phyddle_ms.

## Supplementary Material

Supplement 1
