# Supplementary material for: phyddle: software for phylogenetic model exploration with deep learning
Source: bioRxiv. 2024 Aug 8:2024.08.06.606717. Preprint. [Version 1] doi: 10.1101/2024.08.06.606717 (PMC11326143; doi:10.1101/2024.08.06.606717)
Supplement: Supplementary file 1 [file media-1.pdf]

Version dated: August 6, 2024

Supplementary Information: `phyddle`: simulation-trained deep learning predictions for phylogenetic models

Michael J. Landis[1,*], Ammon Thompson[2]

[1]*Department of Biology, Washington University, St. Louis, MO, 63110, USA*

[2]*Participant in an education program sponsored by U.S. Department of Defense (DOD)*

[*]**Corresponding author:** E-mail: michael.landis@wustl.edu.

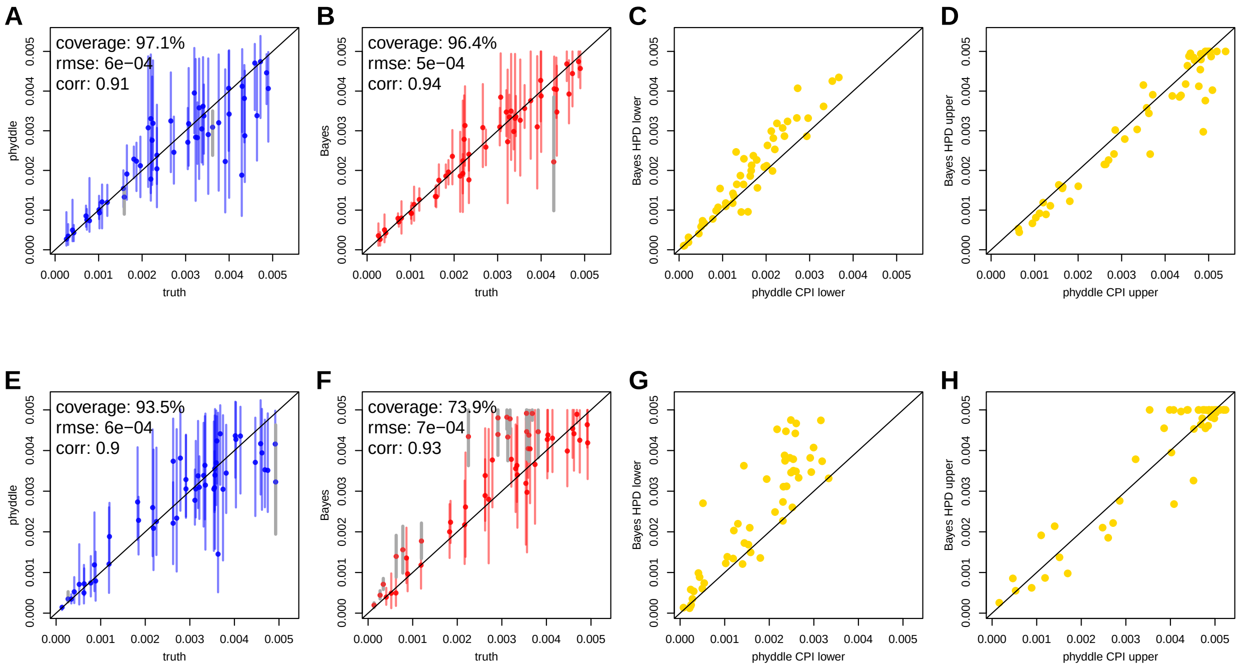

Figure S1: Comparison of Bayesian and `phyddle` estimates for the sampling rate, δ, when all pathogens are sampled during the exponential growth phase of an outbreak (A-D) or sampled at any time during an outbreak (E - H). True parameter values are plotted against `phyddle` (blue; A and E) and Bayesian (red; B and F) point estimates. Estimated support interval bounds (gold; C, D, G, and H) for `phyddle` and Bayesian methods are also plotted against each other. Any point that falls on a slope-1 intercept-0 line has perfectly matching x and y values. Data displayed is a random subsample of 50 values (roughly 50%). Intervals shown are 95% CPI (calibrated probability interval) or HPD (highest posterior density). Bayesian estimates and test data for comparison of exponential phase data (A-D) are from (Thompson et al. 2024). See main text for analysis details (Landis and Thompson 2024).

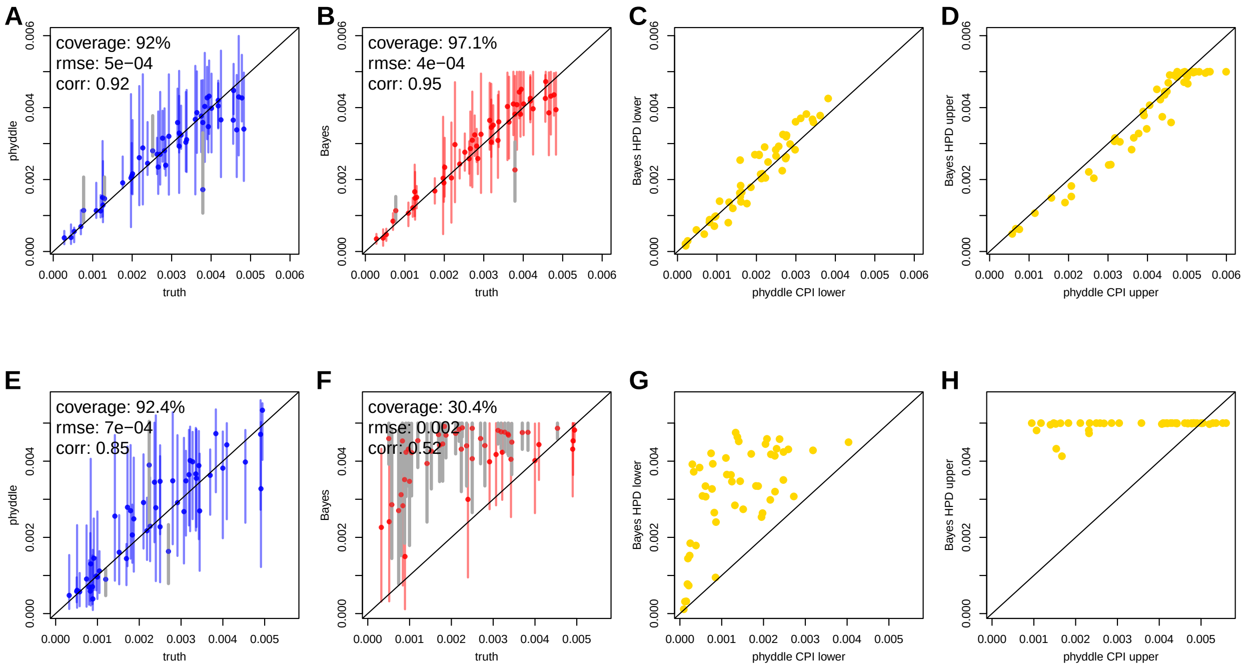

Figure S2: Comparison of Bayesian and `phyddle` estimates for the migration rate, m, when all pathogens are sampled during the exponential growth phase of an outbreak (A-D) or sampled at any time during an outbreak (E - H). True parameter values are plotted against `phyddle` (blue; A and E) and Bayesian (red; B and F) point estimates. Estimated support interval bounds (gold; C, D, G, and H) for `phyddle` and Bayesian methods are also plotted against each other. Any point that falls on a slope-1 intercept-0 line has perfectly matching x and y values. Data displayed is a random subsample of 50 values (roughly 50%). Intervals shown are 95% CPI (calibrated probability interval) or HPD (highest posterior density). Bayesian estimates and test data for comparison of exponential phase data (A-D) are from (Thompson et al. 2024). See main text for analysis details (Landis and Thompson 2024).

*

References

M. J. Landis and A. Thompson. `phyddle`: software for phylogenetic model exploration with deep learning. *bioRxiv*, 2024.

A. Thompson, B. J. Liebeskind, E. J. Scully, and M. J. Landis. Deep learning and likelihood approaches for viral phylogeography converge on the same answers whether the inference model is right or wrong. *Systematic Biology*, 73(1):183–206, 2024.
